# Supplementary material for: PrEP awareness and use among reproductive age women in Miami, Florida
Source: PLoS One. 2023 Jun 7;18(6):e0286071. doi: 10.1371/journal.pone.0286071 (PMC10246803; doi:10.1371/journal.pone.0286071)
Supplement: S1 Table — (DOCX) [file pone.0286071.s001.docx]

| S1 Table. Multivariable Logistic Regression Assessing Predictors of PrEP Awareness (n = 247)^a^ | | |
| --- | --- | --- |
| Parameter | **Adjusted OR (95% CI)** | **p** |
| Race *(reference = White)* |  |  |
| Black | 0.38 (0.15, 0.96) | **0.04** |
| Other | 0.77 (0.29, 2.05) | 0.61 |
| Hispanic *(reference = non-Hispanic)* | 0.18 (0.08, 0.39) | **<0.01** |
| Below Poverty Level | 2.00 (1.04, 3.87) | **0.04** |
| Heterosexual  *(reference = Lesbian, Bisexual, Other)* | 0.29 (0.11, 0.77) | **0.01** |
| Current Diagnosis of Bacterial Vaginosis | 2.28 (1.18, 4.40) | **0.01** |
| Lifetime History of HIV Test | 6.42 (2.83, 14.52) | **<0.01** |
| Condom Use with Male Partners in Past Month *(reference = Always used Condoms)* |  |  |
| Sometimes used Condoms | 0.21 (0.08, 0.56) | **<0.01** |
| Never used Condoms | 0.60 (0.27, 1.31) | 0.20 |
| Number of Male Sexual Partners in past month | 1.30 (1.01, 1.68) | **0.04** |
| *Note.*  ^a^51 observations were not included due to missing values for the outcome or explanatory variables. | | |
